# INTERPRETing to increase access to PSYcho-oncology care (INTERPRET-PSY) for cancer patients with limited English proficiency: study protocol for a pilot randomized controlled trial

**DOI:** 10.1186/s40814-026-01770-5

**Published:** 2026-01-29

**Authors:** Florence Lui, Francesca Gany, Adriana Espinosa, Carlos Riobo, Fennie Chang, Bharat Narang, Ashley Rodriguez, Javier González, Yunshan Niu, Ruo Yan Chen, Rina Jiang, Jennifer Leng

**Affiliations:** 1 Department of Psychiatry and Behavioral Science, Memorial Sloan-Kettering Cancer Center, New York, USA; 2 Memorial Sloan Kettering Cancer Center, New York, USA; 3 CCNY: The City College of New York, New York, USA; 4 Indiana University School of Medicine, Indianapolis, IN USA

**Keywords:** Limited English proficiency, Psycho-oncology, Feasibility studies, Telemedicine, Counseling, Survivorship

## Abstract

**Background:**

People with cancer who have limited English proficiency (LEP) are at high risk for poor mental health outcomes in survivorship as they face cultural, linguistic, and mobility-related barriers to accessing psychosocial care. Telemental health interventions can be of great utility because they can decrease participation burden and are cost-effective. Patients with LEP who receive telemental health services primarily access them through remote interpretation due to a shortage of bilingual therapists. Remote interpreting can be: (1) simultaneous (rarely used in medical settings) or consecutive (usual care in medical settings). Prior research has found that Remote Simultaneous Medical Interpreting (RSMI) resulted in fewer errors, better medical outcomes, and higher patient satisfaction than consecutive methods in oncology and primary care settings. Yet, RSMI has not been studied in the context of a counseling/psychotherapy encounter. INTERPRET-PSY is a mixed method, 3-arm pilot randomized controlled trial (RCT) designed to determine if RSMI is feasible and acceptable in comparison to usual care (RCMI) and “gold standard” care (bilingual provider) in delivering counseling/psychotherapy to Mandarin-speaking LEP cancer patients.

**Methods:**

A total of 45 participants will be randomly assigned to one of 3 arms (*n* = 15 RCMI, *n* = 15 RSMI, and *n* = 15 bilingual provider) to receive 6 counseling/psychotherapy sessions. Feasibility and acceptability of the interpreting modalities (RCMI, RSMI) will be evaluated through self-report assessments. Secondary outcomes will include therapeutic alliance, MCP-Ch content comprehension, and clinically significant interpreting errors across the interpreting (RCMI, RSMI) arms. Semi-structured interviews with a purposively sampled subset of patients, their interventionists, and interpreters (if applicable) across all 3 arms will assess potential barriers and facilitators to successful interpreter-mediated communication and outcomes.

**Discussion:**

INTERPRET-PSY is the first study to examine the feasibility and acceptability of delivering a culturally adapted, evidence-based, and telehealth-delivered counseling/psychotherapy intervention to cancer patients with LEP via RCMI and RSMI. Results of the pilot RCT will be submitted for publication in a peer-reviewed journal and serve as preliminary data for a larger, multisite RCT grant application for a fully powered RCT of interpreting modalities in the provision of linguistically accessible and culturally appropriate psycho-oncology care.

**Trial registration:**

NCT02112188

**Supplementary Information:**

The online version contains supplementary material available at 10.1186/s40814-026-01770-5.

## Background

More than 67 million people in the U.S. speak a language other than English at home and more than 26 million (8% of the population) have limited English proficiency (LEP) [1]. While most people with LEP are immigrants, nearly 19% were born in the U.S., most to immigrant parents [2]. People with LEP are anticipated to reach 19% of the U.S. population by 2050 [1].

This fast-growing population is at disproportionate risk for poor cancer outcomes. Patients with LEP (immigrant or US-born) are less likely to access preventive medical care, such as cancer screenings and regular medical visits [3, 4], due to financial, cultural, and linguistic barriers to healthcare access and navigation [5]. For example, they may delay seeking care to avoid communication difficulties, out of concern about the costs of medications or copayments, or because they doubt their needs will be adequately addressed by healthcare providers [6]. These disparities in preventive care contribute to higher cancer mortality rates, as suggested by a meta-analysis that found immigrant women were more likely to present with breast cancer at an advanced stage than native-born women [7]. Although the meta-analysis did not examine language as a factor in stage at diagnosis, findings indicated that immigrants from countries with developed economies experienced the most similar stage distribution to native-born populations, suggesting immigrants from developing nations—who are also more likely to have LEP—may face more financial or access-related barriers to care than those from developed nations [7]. Indeed, a study of physicians treating LEP breast cancer patients found 75% of physicians reported using untrained interpreters [8], which has been shown to result in more errors of clinical consequence [9]. Given these disparities in cancer outcomes and care, individuals with LEP may be especially susceptible to poor psychosocial outcomes in survivorship. Research indicates that foreign-born, ethnoracial minority, LEP, and lower-income cancer patients are more likely to report higher levels of distress and poorer quality of life than native-born, non-Hispanic White, English proficient, and higher-income patients [10–12].

Psychosocial interventions show significant promise in improving quality of life and emotional adjustment outcomes for cancer patients. However, the vast majority of intervention trials have been conducted in predominantly non-Hispanic White (NHW) English-speaking, and middle-to-high income samples [13]. Our systematic review and meta-analysis of psychosocial interventions for immigrant and LEP cancer patients found only 9 psychotherapy/counseling interventions available to non-English speakers. Of these, only 3 were delivered by telephone, despite our finding that in-person and telephone-delivered interventions were equally effective [14]. Given the potential physical mobility issues that all cancer patients face, as well as competing time burdens and systemic barriers to accessing mental health care common among immigrant and LEP patients, telemental health interventions, which can decrease participation burden and are typically more cost-effective to implement, may be of great utility in this population [15, 16]. Past research suggests high telehealth engagement potential among LEP patients, with rates of intervention adoption over 65% when they were specifically tailored for LEP patients [17, 18].

The majority of patients with LEP who access mental health care do so through medical interpreters, given a national shortage of bilingual mental health professionals [19]. Typically, medical interpretation is consecutive (interpreting is provided once a speaker has finished speaking) [20]. RSMI or “United Nations-style” interpreting is similar to a voice-over and is provided remotely by trained medical interpreters [20]. RSMI is likely superior to consecutive interpreting methods due to increased efficiency [21, 22], fewer clinically significant medical errors [21, 22], and enabling a more private and dyadic clinical encounter [21, 23]. By contrast, the most commonly employed interpreting strategy, Remote Consecutive Medical Interpreting (RCMI), can interfere with the collaborative relationship between patient and therapist (i.e., therapeutic alliance) [24], one of the most robust predictors of psychosocial intervention outcomes [25]. In addition, consecutive interpreting can impede the natural flow of communication, as interpreters pace their interlocutors. Yet to our knowledge, RSMI has not yet been studied in the telemental health context; a systematic review on the use of interpreters in mental health found no quantitative studies on interpreter accuracy nor on the influence of interpretation modes on the therapist–patient relationship [26].

To address these limitations, INTERPRET-PSY is a mixed method, explanatory sequential (QUANT qual), 3-arm pilot randomized controlled trial (RCT) developed to determine whether RSMI is feasible and acceptable in comparison to usual care (RCMI) and “gold standard” care (bilingual provider) in delivering psycho-oncology counseling to Mandarin-speaking Chinese cancer patients with LEP.

## Methods

### Overview and theoretical framework

**Fig. 1:**
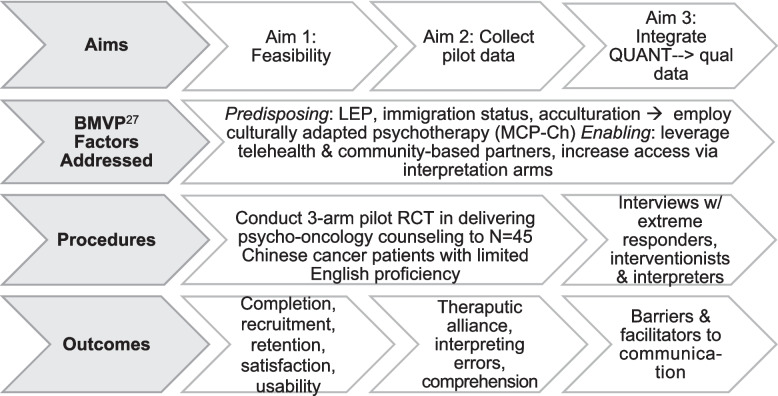
Aims, procedures, outcomes, and theoretical framework of the INTERPRET-PSY study

The study is guided by the Behavioral Model for Vulnerable Populations (BMVP), which identifies factors that influence the health-seeking behavior of “vulnerable” or medically underserved populations [27]. Predisposing factors such as limited English proficiency, immigration status, and acculturation level may lead to a decrease in seeking healthcare. Enabling factors, such as the availability of linguistically appropriate care and community resources, facilitate health-seeking behaviors. Our study seeks to address predisposing factors and maximize enabling factors to increase access to needed psychosocial care among cancer patients with LEP (Fig. 1).

The methods for INTERPRET-PSY were developed in accordance with the Standard Protocol Items: Recommendations for International Trials (SPIRIT) guidelines [28]. Any prospective amendments to the protocol, eligibility, or outcomes will first be approved by MSK’s Institutional Review Board.

### Key study objectives

1. Assess the feasibility of interpreting modes in delivering counseling sessions to Chinese cancer patients with LEP. RSMI and RCMI feasibility will be determined by the completion of ≥5 out of 6 sessions for *n* = 11 per arm (*primary outcome*). We will also collect descriptive data on recruitment/randomization/retention rates; acceptability (*secondary outcome*); and telehealth usability for all 3 arms.
2. Collect pilot data on outcomes central to RSMI and RCMI-enabled psycho-oncology counseling. We will summarize post-intervention scores on therapeutic alliance (Working Alliance Inventory), treatment expectancy (i.e., the degree to which participants expect a good outcome from counseling), and counseling content comprehension by arm (RSMI, RCMI, bilingual provider), and will compare the number of clinically significant interpreting errors in MCP-Ch Session 1 across RSMI and RCMI arms *(secondary outcomes)*. We will also summarize post-intervention scores on psychosocial outcomes (e.g., distress, spiritual well-being, quality of life, anxiety and depression, meaning in life, and hopelessness) by arm (*exploratory outcomes*).
3. To inform “best practices” in telehealth delivery of interpreter-mediated psycho-oncology care. We will complement quantitative outcome data with qualitative data from semi-structured interviews with *n*~ 8 “extreme responders” (~6 per arm, pending saturation) and their interventionists and interpreters (if applicable). Interviews will contextualize outcomes and identify barriers and facilitators to interpreter-mediated communication in counseling.

### Trial design

**Fig. 2:**
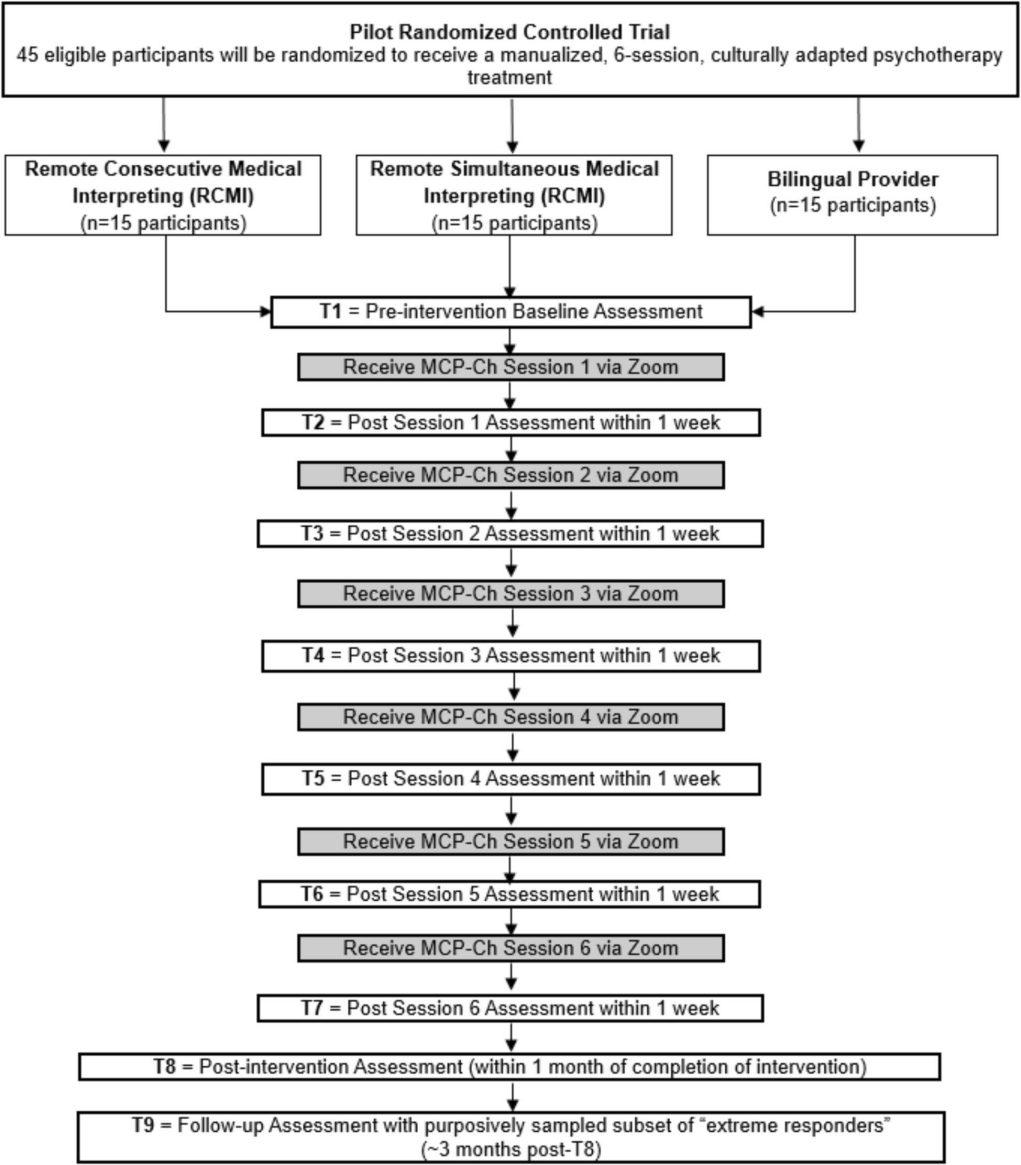
INTERPRET-PSY Pilot RCT Timeline

INTERPRET-PSY is a mixed method, explanatory sequential (QUANT qual), 3-arm pilot randomized controlled trial (RCT) being conducted in preparation for a subsequent, fully powered RCT evaluating the efficacy of interpreting methods to deliver psycho-oncology care. A timeline of the INTERPRET-PSY project is presented in Fig. 2. This open-label, parallel-group RCT aims to enroll 45 Mandarin-speaking patients with LEP. Participants will be registered by the Memorial Sloan Kettering Cancer Center (MSK) institution-wide, central Protocol Participant Registration system and randomized (using the Clinical Research Database) with equal probability to the RSMI, RCMI, or bilingual provider group and will complete self-report measures (*n* = 15 in each group). As this is a pilot study and not a fully powered RCT, the sample size was determined based on resource considerations and a sample size calculation for the primary outcome of RSMI/RCMI feasibility (see Data analysis plan below) [29]. A purposively sampled subset of participants (i.e., high- and non-responders) in each arm (*n*~6 per arm, pending saturation) and their interpreters and interventionists will be followed up for qualitative data collection in semi-structured interviews to contextualize outcomes and identify barriers and facilitators to interpreter-mediated psycho-oncology counseling.

### Location and participants

The study will take place at MSK in New York City. Research assistants will screen potential patients from MSK and self-referring patients via community providers and consent them as research participants following approval from their primary oncologists. Additionally, a subset of purposively sampled participants as well as their interpreters and interventionists will be consulted through exit interviews to identify barriers and facilitators to successful delivery of MCP-Ch via remote interpreting. All research participants will be compensated to promote retention and complete follow-up. Patients will receive compensation of $50 following completion of each assessment and exit interview. Purposively sampled participants who complete the T3 assessment, their interpreters, and interventionists will receive $50 following completion of the semi-structured interview.

### Eligibility criteria

Inclusion criteria:

1. Speaks English less than “very well” (i.e., has limited English proficiency)
2. Prefers to speak in Mandarin Chinese
3.  ≥18 years of age
4. of Chinese descent
5. Diagnosis of stage III or IV cancer (or roughly equivalent for non-solid tumor cancers)
6. Resides in New York or New Jersey (where the PI is licensed)

Exclusion criteria:

1. Unable/unwilling to use the Zoom platform for telehealth delivery and remote interpretation (*Note*: Participants without an Internet-enabled device will be given or loaned one, and research staff will be available to assist with accessing, downloading, and troubleshooting Zoom if needed).
2. Diagnosed with a primary brain tumor
3. Currently undergoing hematopoietic stem cell transplantation (HSCT)
4. Major cognitive impairment that would preclude study participation

## Interventions

### RSMI, RCMI, or bilingual provider

Participants assigned to the (1) RSMI, (2) RCMI, and (3) bilingual provider arms (*n* = 15 per arm) will receive all 6 counseling sessions (for details on these sessions, see below) via an English-speaking interventionist and a RSMI interpreter (i.e., a trained bilingual Mandarin-English interpreter who provides remote interpretation within milliseconds of the original speech, nearly simultaneously, almost like a voiceover); an English-speaking interventionist and a RCMI interpreter (i.e., a trained bilingual Mandarin-English interpreter who provides remote consecutive interpretation—by listening as the primary speaker speaks and interpreting only once the primary speaker has finished); and a bilingual interventionist in Mandarin, respectively. Sessions for all 3 arms will take place using a videoconferencing platform and will be recorded. To promote adherence and retention, participants will receive session reminders 1 week, 3 days, and the day prior to the appointment by phone, text, and/or email (with a link to the videoconferencing platform); participants who cannot attend will be given an option to reschedule. Mandarin-English interpreters will be certified as having native bilingual fluency (speaking, understanding, reading, and writing) and meet performance standards for both consecutive and simultaneous interpreting, since they will serve as both RSMI and RCMI interpreters. They will receive intensive training on professional standards of practice and ethics, medical terminology, and linguistic/cultural responsiveness, and will be required to meet professional interpreter standards before study interpreting.

### Description of counseling sessions

The six counseling sessions will utilize the Meaning-in-life Counseling Program for Chinese cancer patients (MCP-Ch) [30], a cultural and linguistic adaptation [30–32] of Meaning-Centered Psychotherapy (MCP). Meaning-Centered Psychotherapy (MCP) is a brief, structured, manualized intervention grounded in the work of Viktor Frankl [33]. MCP has demonstrated efficacy in enhancing advanced cancer patients’ sense of meaning-in-life and QOL while decreasing feelings of depression, anxiety, desire for hastened death, and distress in several randomized controlled trials [34–36]; for a conceptual framework of MCP, see Breitbart, 2016 [37]. MCP guides patients on how to access “sources of meaning” during challenging times: (1) historical (significant memories, relationships, and traditions and their impact on one’s sense of meaning), (2) attitudinal (confronting limitations imposed by cancer), (3) creative (engaging in life through one’s pursuits, including work, hobbies, life roles), and (4) experiential (connecting with life through experiences with love, beauty, humor, etc.).

MCP-Ch consists of six 60-min individual sessions delivered over the span of 3–12 weeks, depending on participant availability. Adaptations to MCP for the Chinese population focused on changing content and strategies to reflect the Chinese cultural context (Table 1) [30, 38]. MCP-Ch will be delivered by doctoral student clinician interventionists who will receive intensive training prior to delivering the intervention and regular supervision after each session.

**Table 1:** MCP-Ch overview and adaptations

1	Intro to MCP-Ch	Added additional orientation about psychotherapy and addressed confidentiality concerns among undocumented patients; created handouts with MCP-Ch structure and educational information/FAQ to address cancer myths, cancer stigma, and low health literacy
2	Who Am I? Before Cancer and Now	Given Chinese collectivistic cultural values, clarified that one’s identity includes relationships, beliefs, values
3	Your Life Story: Past, Present, and Future	Given death taboo in Chinese culture, replaced “legacy” with “how you wish to be remembered”; added a list of culturally appropriate questions to guide patients in sharing their life story with others
4	Your Attitude toward Life Challenges	Given death taboo and family-centered model of care in China, made question on “What is a good death” optional based on pt’s prognostic awareness and openness to end-of-life discussion
5	Meaning through Living Your Life	Combined 2 sessions into 1 to improve acceptability & comprehension; revised exercise with culturally syntonic examples of love, beauty, and humor
6	Transitions	No major changes based on stakeholder feedback
All sessions		
Used plain language for manual content and exercises, used “think aloud” technique for MCP definitions, and renamed to “Meaning-in-Life Counseling Program” to counter mental health stigma		

### Assignment of interventions

Participants will be registered by the MSK institution-wide, central Protocol Participant Registration system and randomized (using the Clinical Research Database) to receive MCP-Ch with equal probability via (1) an English-speaking interventionist and an RSMI interpreter; (2) an English-speaking interventionist and an RCMI interpreter; or (3) a bilingual Mandarin-speaking interventionist (no interpreter). Research assistants will randomize a participant following the participant’s completion of the consent, eligibility screener, and baseline assessment. Because a PI (Dr. Lui) will be conducting supervision for the interventionists, and because different assessments will be administered depending on the intervention assignment (e.g., clinically significant interpreting errors will be assessed only in the RCMI and RSMI arms), the only person completely blinded to group assignment will be the data analyst/statistician.

## Outcomes

1. The first goal of INTERPRET-PSY is to determine the feasibility of using RSMI and RCMI to deliver a manualized, culturally adapted counseling intervention to LEP cancer patients. Participants will be deemed not evaluable for feasibility if they drop out prior to Session 6 due to non-intervention related issues (e.g., progression of cancer or death). These non-evaluable participants will be replaced and reasons for dropout tracked. A determination of feasibility for RSMI/RCMI (*primary outcome*) will be met if *n* = 11 complete treatment (a comparable 67% MCP completion rate was achieved in Dr. Breitbart’s original MCP trial [35]), defined as completing 5 out of 6 sessions (i.e., ≥11 out of 15 participants completing ≥5/6 sessions). We will also calculate the observed recruitment rate, retention rate, participant satisfaction with treatment protocol (*secondary outcome*, scored by calculating the percentage of positive “agree” responses on a satisfaction survey), and telehealth usability in all 3 arms (RSMI, RCMI, bilingual provider). These outcomes will be measured quantitatively within 1 month following the intervention (T8) through a post-intervention assessment.
2. INTERPRET-PSY also will assess the effect of interpreter-mediated delivery on counseling/psychotherapy process outcomes. This will be measured by comparing the RSMI and RCMI groups to the “gold standard” bilingual provider group at post-intervention assessment (T8), administered within 1 month following the intervention. Outcomes will include therapeutic alliance, MCP-Ch content comprehension, and the number and nature of clinically significant interpreting errors in MCP-Ch Session 1 (*secondary outcomes*); participants’ treatment expectancy, post MCP-Ch session 1 (T2); and distress, spiritual well-being, quality of life, anxiety and depression, meaning in life, and hopelessness (*exploratory outcomes*). We will also assess post-session ratings after each MCP-Ch session (T2, T3, T4, T5, T6, and T7).
3. We will identify barriers and facilitators to successful interpreter-mediated delivery of MCP-Ch in semi-structured interviews with “extreme responders” in each of the 3 arms (i.e., participants who score especially high or low on therapeutic alliance, MCP-Ch comprehension, and/or acceptability). These will be assessed approximately 3 months post-intervention (T9).

## Measures

A description of each quantitative measure used at each assessment is provided in Table 2, and a timeline of assessments is provided in Table 3. Most measures have demonstrated good reliability and validity in Mandarin-speaking populations (Table 2). Surveys that do not have validated Chinese translations will be translated and checked for quality by qualified translators using an established five-step process for translation and evaluation developed by Forsyth et al. [39]. All measures will be administered either by phone or videoconference by trained bilingual research staff and entered into REDCap, a secure web platform for electronic data capture.

**Table 2:** List of assessments

Measure Title	Time Points Used	Outcome	Description of Measure
Demographic and Clinical Characteristics	T1 (Baseline)	n/a	Age (years), gender, race, ethnicity, education, preferred language, level of English proficiency, country of origin, immigration length, frequency of revisiting birthplace, employment status, household income, marital status, household size, religious/spiritual beliefs, health insurance status, most recent cancer diagnosis, and current cancer treatment received
Digital Health Care Literacy (DHCL) [40]	T1 (Baseline)	n/a	The DHCL is a brief (3-item), face valid measure of one’s confidence in the ability to use technological programs or services and the ability to independently troubleshoot technical issues. Cronbach’s α = 0.90 [36]
Feasibility	T8 (Post-intervention)	Primary	Feasibility will be assessed via attendance logs and defined as ≥11 out of 15 participants completing ≥5/6 sessions
Acceptability	T8 (Post-intervention)	Secondary	A 20-item, investigator-initiated, face-valid survey to assess participants’ satisfaction with the intervention, mode of delivery (via RSMI or bilingual interventionist) and effects of treatment (i.e., whether the intervention was helpful). Acceptability will be scored by calculating the percentage of positive (“agree”) responses
Therapeutic Alliance (WAI-S) [41]	T2, T3, T4, T5, T6, T7 (Post-sessions 1–6)	Secondary	The Working Alliance Inventory-Short Form (WAI-S) is a 12-item self-report scale of therapeutic alliance with 2 forms: one for the client, and one for the interventionist. Cronbach’s α = 0.86. [42]*
MCP-Ch Content Comprehension	T8 (Post-intervention)	Secondary	A 10-item, face-valid, investigator-initiated questionnaire. These multiple-choice items assess a participant’s understanding of MCP concepts presented in the MCP-Ch program
Clinically significant interpreting errors in MCP-Ch Session 1	T8 (Post-intervention)	Secondary	The number and nature of clinically significant interpreting errors made in MCP-Ch Session 1 (RCMI, RSMI arms only) will be assessed using an error analysis tool developed by Gany et al. [21] For more details, see Data analysis plan, objective #2
Credibility/Expectancy Questionnaire (CEQ) [43]	T2 (Post-session 1)	Exploratory	The CEQ is a 6-item questionnaire, half of which relates to the subscale of credibility (i.e., how believable, convincing, and logical the treatment is) and the other half to expectancy (i.e., the improvements the patient believes will be achieved). *r* = 0.85. [49]
Distress Thermometer (DT) [44]	T1 (Baseline), T8 (Post-intervention)	Exploratory	The Distress Thermometer (DT) is a single-item scale used to screen cancer patients for the presence of psychological distress with a 0–10 range. *r* = 0.80 [45].*
Spiritual Well-Being Scale (FACIT-Sp) [46]	T1 (Baseline), T8 (Post-intervention)	Exploratory	The Functional Assessment of Chronic Illness Therapy—Spiritual Well-Being Scale (FACIT-Sp) is a 12-item self-report instrument assessing the spiritual well-being of people with chronic and/or life-threatening illnesses. It has three subscales: Peace, Meaning, and Faith. Cronbach’s α = 0.89 [47]*
McGill Quality of Life Questionnaire (MQOL) [48]	T1 (Baseline), T8 (Post-intervention)	Exploratory	The McGill Quality of Life Scale (MQOL) is a 16-item quality of life self-report measure for people with life-threatening illness. It is composed of 4 subscales: physical symptoms, psychological symptoms, outlook on life, and meaningful existence. Cronbach’s α = 0.87 [49]*
Hospital Anxiety and Depression Scale (HADS) [50]	T1 (Baseline), T8 (Post-intervention)	Exploratory	The 14-item Hospital Anxiety and Depression Scale (HADS) measures both anxiety and depression symptoms. Cronbach’s α = 0.93 [51]*
Meaning in Life Questionnaire (MLQ) [52]	T1 (Baseline), T8 (Post-intervention)	Exploratory	The MLQ is a 10-item measure of meaning-in-life with 2 factors: Presence and Search. Cronbach’s α = 0.84-0.88 depending on subscale [53].*
Beck Hopelessness Scale (BHS) [54]	T1 (Baseline), T8 (Post-intervention)	Exploratory	The Beck Hopelessness Scale (BHS) is a 20-item true–false self-report measure of participants’ degree of pessimism and hopelessness. Cronbach’s α = 0.85 [55].*
Weekly Session Rating and Telehealth Evaluation	T2, T3, T4, T5, T6, T7 (Post-sessions 1–6)	n/a	A 10-item investigator-initiated session rating and telehealth evaluation assessing participant impressions of each intervention session, as well as any technical difficulties with videoconferencing or telephone delivery
Telehealth Usability (TSQ) [56]	T8 (Post-intervention)	n/a	The 14-item Telemedicine Satisfaction Questionnaire evaluates patient-reported usefulness, ease of use, effectiveness, reliability, and satisfaction of how telehealth delivery of an intervention functioned in comparison to a traditional in-person encounter. Cronbach’s α = 0.93 [52]
Exit Survey	T8 (Post-intervention)	n/a	A 4-item, investigator-initiated survey to collect participant feedback for program improvement

*Reliability statistics from studies of validated Chinese translations

**Table 3:**
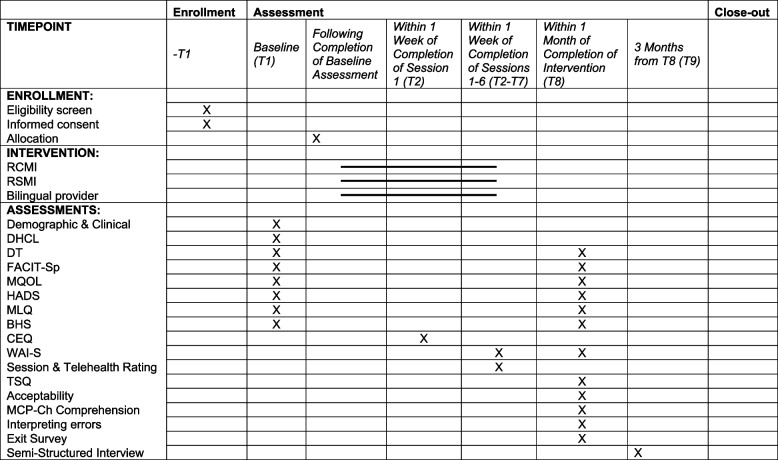
SPIRIT timeline of assessments

Standard Protocol Items: Recommendations for Interventional Trials (SPIRIT) figure. *DHCL* Digital Health Care Literacy, *DT* distress thermometer, *FACIT-Sp* Functional Assessment of Chronic Illness Therapy - Spiritual Well-Being, *MQOL* McGill Quality of Life Questionnaire, *HADS* Hospital Anxiety and Depression Scale, *MLQ* Meaning in Life Questionnaire, *BHS* Beck Hopelessness Scale, *CEQ* Credibility/Expectancy Questionnaire, *WAI-S* Working Alliance Inventory-Short Form, *TSQ* Telehealth Satisfaction Questionnaire

### Screener

Twelve self-report items will assess eligibility (e.g., level of English proficiency, preferred language, age, Chinese ethnicity, cancer diagnosis and stage, and state of residence). In addition, 3 items (i.e., cancer diagnosis and stage, HSCT, and presence of cognitive impairment) require study staff to screen participants’ electronic medical record and, if needed, follow up with the patient’s physician for confirmation of eligibility.

### Pre-intervention/baseline assessment (T1)

Patients will be asked the following demographic and clinical characteristics: their age (years), gender, race, ethnicity, education, preferred language, level of English proficiency, country of origin, immigration length, frequency of revisiting birthplace, employment status, household income, marital status, household size, religious/spiritual beliefs, health insurance status, most recent cancer diagnosis, and current cancer treatment received. Participants will also respond to the following: Digital Health Care Literacy (DHCL) [40], Distress Thermometer (DT) [44], Spiritual Well-Being (FACIT-Sp) [46], Quality of Life (MQOL) [48], Anxiety and Depression (HADS) [50], Meaning in Life (MLQ) [52], and Beck Hopelessness Scale (BHS) [54]. 

### Post-session 1 only (T2)

Credibility/Expectancy Questionnaire (CEQ) [43]

### Post-session assessments (T2, T3, T4, T5, T6, T7)

Therapeutic Alliance (WAI-S) [41], Weekly Session Rating and Telehealth Evaluation

### Post-intervention assessment (T8) administered within 1-month post-intervention

Distress Thermometer (DT) [44], Spiritual Well-Being (FACIT-Sp) [46], Quality of Life (MQOL) [48], Anxiety and Depression (HADS) [50], Meaning in Life (MLQ) [52], Beck Hopelessness Scale (BHS) [54], Telehealth Usability (TSQ) [56], Acceptability, MCP-Ch Concept Comprehension, and Exit Survey

### Qualitative interviews (T9)

Approximately 27 (or until saturation) INTERPRET-PSY trial participants, interpreters, and interventionists will be invited to complete semi-structured interviews with study staff, via teleconference or phone. We estimate that interviewing ~6 participants per arm (~18 patients/participants), ~3 interpreters, and ~6 interventionists will be sufficient based on prior interview-based studies indicating metathemes can be identified in as early as 6 interviews and thematic saturation within 12 interviews [57]. Interviews will be conducted in Mandarin with patients/trial participants, in English with interventionists, and in Mandarin or English with interpreters (based on interpreter preference). The interviews will be approximately 45 min. Interviews will be conducted by study staff using a semi-structured guide with the following domains: (1) general feedback about their experiences with the INTERPRET-PSY study (i.e., for *patients/participants*, what they thought of the Mandarin-speaking abilities of their interventionist and/or interpreter; perceived relationship/rapport with their interventionist and/or interpreter; and their preferences for consecutive vs. simultaneous interpretation; for *interpreters*, their experience providing interpreting services and preferences for consecutive vs. simultaneous interpretation; for *interventionists*, their experience conducting psycho-oncology counseling and their preferences for consecutive vs. simultaneous interpretation) and (2) barriers and facilitators to remote interpreting implementation in the delivery of psycho-oncology care (i.e., barriers and facilitators to interpreter-mediated communication, the development of therapeutic alliance, and intervention delivery). To facilitate mixed methods integration, selection of patients/trial participants will be guided by quantitative results (i.e., low vs. high therapeutic alliance, low vs. high intervention comprehension, low vs. high acceptability) and interview questions soliciting barriers and facilitators will contain exploratory probes based on scores. For example, for patients/participants scoring low on comprehension, “What were some barriers in the communication process that may have made it difficult understand concepts in the Meaning-in-Life Counseling Program?”.

### Data analysis plan (by objective)

1. Counseling completion rates will be calculated; we will treat each interpreting arm (RSMI, RCMI) as a single stage design and will assess each separately for feasibility (*primary outcome*). We will calculate frequencies and percentages to summarize recruitment, randomization, retention, acceptability (*secondary outcome*), and usability across all 3 arms (RSMI, RCMI, and bilingual provider). Based on the calculation outlined by Khan, Sarker, and Hackshaw [58], observing 11 or more successes out of 15 in a given arm would allow us to be sufficiently confident that the population feasibility of the corresponding interpretation mode is 90% against a null of 40%. In our calculations, we have set our statistical power to 90% and our Type I error rate to 0.025 to reduce the chance of false discovery. This provides us with sufficient combined statistical power, assuming the two tests are independent.
2. We will summarize (1) therapeutic alliance scores and MCP-Ch content comprehension at T4 across all 3 arms (RSMI, RCMI, and bilingual provider) and (2) number and nature of clinically significant interpreting errors in MCP-Ch Session 1 across the interpreting arms (RSMI, RCMI) (*secondary outcomes*). We will also summarize treatment credibility/expectancy at T2 and differences in post- vs. pre-intervention distress, spiritual well-being, quality of life, anxiety and depression, meaning-in-life, hopelessness by arm (*exploratory outcomes*). These descriptive summaries by arm will provide preliminary indications of whether delivery of psycho-oncology counseling via RSMI or RCMI may potentially achieve clinically significant benefits, which will aid in the design of, and be further assessed in, a subsequent fully powered trial [29]. We will assess baseline contextual factors at T1 (e.g., digital health care literacy, level of English proficiency) to examine whether therapeutic alliance and MCP-Ch content comprehension scores differ by these variables. We will use an error analysis tool developed by Gany et al. [21] to obtain the number and nature of clinically significant errors in MCP-Ch Session 1. Specifically, a panel of 2–4 psycho-oncology clinicians blinded to the interpreting method (i.e., RSMI or RCMI) will review and code transcripts. They will independently code each script to identify clinically significant content and meet to reconcile any differences in their selection of clinically significant content. Examples of potentially clinically significant content include history of cancer diagnosis, past psychiatric history, MCP-Ch session exercises, and plans for future visits. Once all clinically significant content has been identified, a second blinded panel of 2–4 bilingual coders will independently code clinically significant content to identify interpreting errors, yielding a total count of clinically significant interpreting errors. To ensure rigor, coders on both panels will first separately conduct preliminary analyses on a subset of transcripts using the described protocol. Interrater reliability will be assessed by calculating percent agreement. Consensus meetings will be held to adjudicate disagreements and refine error categories and the analytic approach as needed until strong interrater reliability is reached (i.e., *k* = 0.80), at which point coders will commence coding of the remaining transcripts.
3. To assess our semi-structured interviews with “extreme responders,” we will use thematic content analysis [59] to derive insights directly from participants’ narratives, following established guidelines for conducting rigorous qualitative research [60]. First, coders will each read a subset of interviews and develop codes to characterize responses. Coders will then meet to establish an initial codebook, which will be used to code the remaining interviews. Regular consensus meetings will be held to confirm the reliability of coding. The codebook will be iteratively revised as new codes emerge, and codes will be summarized into themes. Prominent themes will include those observed across the majority of interviews as well as key divergences between sub-groups. Findings will be integrated using NVivo [61], which enables qualitative and quantitative data to be paired by participant and in summary format. The coded qualitative data will be explored in relationship to participant outcomes, baseline characteristics, contextual factors, and study arm to further elucidate barriers and facilitators to the implementation of remote interpreting in the psycho-oncology setting.

### Data and safety monitoring

The Data and Safety Monitoring Plan will align with the MSK DSM Plan where possible: https://one.mskcc.org/sites/pub/clinresearch/Documents/MSKCC%20Data%20and%20Safety%20Monitoring%20Plans.pdf and MSK’s Data Safety and Monitoring Committee (DSMC) will have oversight of the trial, including protocol monitoring, compliance, and data verification audits, staff education on clinical research quality assurance. The DSMC will initiate review following the enrollment of the first participant, or by the end of year one if no accruals, and will continue for the study lifecycle until there are no participants under active therapy and the protocol has closed to accrual.

### Adverse reactions and events

Participants may find some questions in the psychosocial assessments upsetting. However, as measures were chosen to reflect what are likely to be existing concerns, the present study is not expected to markedly increase participants’ psychological distress above their routine concerns. Topics covered during MCP-Ch sessions may be emotional, but related distress is expected to be transient and will be supported by a mental health clinician. In addition, experienced research staff trained in interviewing medically ill individuals will administer all study measures and will be supervised by the study principal investigators (PIs). If a participant wishes to stop participating in the study for any reason, we will request that they inform the research team and, if willing and able, their reason for ceasing participation. If a participant appears to be at risk for harming himself/herself or others during the course of the trial, the researchers will take immediate action to address this risk and the participant would become ineligible for continuing with the study.

Potential adverse events for this trial are expected to be non-physical in nature. The principal investigators will report unanticipated and serious adverse events to the IRB in a timely manner on an ongoing basis. For the purposes of this study, a serious adverse event is defined as an event that, as a direct result of the study, causes serious harm (e.g., death or serious injury) to the participant. Adverse events are also reported as part of the progress reports submitted to the National Institutes of Health. If at any point during the study period the intervention is found to be associated with an undue risk of harm to participants, then the trial will be stopped—such as if the research team determines, in good faith, that the intervention appears to be causing significant emotional distress or impairment for participants beyond what would be expected or leading to increased risk for harm to self or others.

### Protection of human subjects

All study staff involved in the research are educated on the protection of human research participants, and the proposed research will comply with the regulations described in 45 CFR Part 46, Protection of Human Subjects. All personnel involved in the proposed protocol have been educated regarding HIPAA regulations and fully understand their responsibilities to safeguard the personal health information of every participant involved in the research. Any participant participating in the study may decline to continue participation and may withdraw from the study at any time. Any participant who expresses a desire for more psychosocial support following the intervention will receive a customized set of referrals from the study team. If research staff identify signs indicating a significant and acute risk of harm to self or others, such information will immediately be shared with the PIs of the study so that a plan can be enacted for timely and appropriate assessment and care, provided by a licensed/board-certified mental health provider or local clinicians (e.g., emergency rooms near the study participant). Participant confidentiality will only be broken if information gathered during the study indicates that the participant poses a significant and acute risk of harm to self or others. Prior to inclusion in the study, participants will be informed of this exception as part of the informed consent process.

## Discussion

This trial will evaluate the feasibility and acceptability of delivering psycho-oncology counseling to Chinese cancer patients with LEP using RSMI vs. RCMI (usual care) vs. the “gold standard” bilingual provider. Psychotherapy process outcomes (therapeutic alliance, intervention comprehension, number of clinically significant interpreting errors) as well as psychosocial outcomes will be examined descriptively by arm.

In accordance with best practices for pilot and feasibility trials, this protocol is designed to assess feasibility and inform the design of a future, fully powered trial [29]. We have defined our primary outcome, feasibility, as observing 11 out of 15 successes (i.e., at least 11 participants out of 15 completing 5 of 6 sessions per arm). To determine if progression from our pilot trial to a future definitive trial is warranted, we will follow Mellor et al.’s “traffic light” set of progression criteria [62]: we will proceed/go (green) to the definitive trial if we observe ≥11 out of 15 successes per arm, amend (yellow) our design to improve feasibility of the methods or design if we observe 7–10 successes per arm, and will stop/not proceed (red) if we observe 6 or fewer successes per arm. We will additionally explore whether RSMI can achieve clinically significant changes to a clinical outcome of interest, quality of life (*exploratory outcome*), defined as an improvement of 1.5 points or greater on the MQOL score [63].

We acknowledge several limitations of the study protocol. First, due to the clinical and logistical aspects of the protocol, the INTERPRET-PSY trial will not be double blinded. Second, this pilot RCT has limited statistical power and insufficient statistical power to evaluate the effectiveness of the intervention. To address this, future studies should include a larger sample size to increase statistical power and the reliability of results. As recommended by Kraemer et al. [64] however, we intend to use feasibility indicators as well as the minimal clinically important difference to inform the design of a large-scale, adequately powered study. Third, the lack of long-term follow-up assessments in this pilot study limits our ability to assess the sustainability of any effects on clinical outcomes of interest, including quality of life, anxiety and depression, and spiritual well-being. We plan to incorporate follow-up assessments beyond the intervention endpoint in a future definitive trial to understand the durability and long-term outcomes of interpreter-assisted psycho-oncology counseling.

To our knowledge, this is the first trial to examine RSMI in the telemental health context. Previous studies have suggested that RSMI may be more efficient, more accurate, and enable a more dyadic clinical encounter between the patient and provider than more commonly employed consecutive interpreting methods [21, 22]. Additionally, the trial’s mixed methods approach, informed by the Behavioral Model for Vulnerable Populations [27], is designed to identify enabling factors to facilitate mental health-seeking behaviors among LEP patients, with the goal of yielding informative “best practices” for mental health practitioners working with interpreters to treat patients with LEP. If RSMI-assisted counseling proves efficacious in a future, fully powered trial, it has the potential to impact clinical practice and programs to mitigate language-based disparities in the provision of mental health services for patients with LEP.

### Trial status

Enrollment for the INTERPRET-PSY trial began in March 2024.

## Supplementary Information

Additional file 1. SPIRIT Checklist.

## Data Availability

The data that will support the findings of this study are available from the corresponding author [FL], upon reasonable request.

## Abbreviations

INTERPRET-PSY: INTERPRETing to increase access to PSYcho-oncology care
LEP: Limited English proficiency
RCMI: Remote Consecutive Medical Interpreting
RSMI: Remote Simultaneous Medical Interpreting
RCT: Randomized controlled trial
NHW: Non-Hispanic White
SPIRIT: Standard Protocol Items: Recommendations for International Trials (SPIRIT)
MSK: Memorial Sloan Kettering Cancer Center
HSCT: Hematopoietic stem cell transplantation
MCP-Ch: Meaning-in-life Counseling Program for Chinese cancer patients
MCP: Meaning-Centered Psychotherapy
DHCL: Digital Health Care Literacy
DT: Distress Thermometer
FACIT-Sp: Functional Assessment of Chronic Illness Therapy - Spiritual Well-Being
MQOL: McGill Quality of Life Questionnaire
HADS: Hospital Anxiety and Depression Scale
MLQ: Meaning in Life Questionnaire
BHS: Beck Hopelessness Scale
WAI-S: Working Alliance Inventory-Short Form
TSQ: Telehealth Satisfaction Questionnaire
